# Efficacy and safety of Chinese herbal medicine Wuwei Xiaodu Drink for wound infection: A protocol for systematic review and meta-analysis

**DOI:** 10.1097/MD.0000000000032135

**Published:** 2022-12-02

**Authors:** Jie Luo, Jingfan Yang, Ming Peng, Fang Liu, Xing Zhou, Hong Yin, Jinlei Li

**Affiliations:** a Taizhou Traditional Chinese Medicine Hospital, Taizhou City, Zhejiang Province, China; b Kunming Municipal Hospital of Traditional Chinese Medicine, Kunming City, Yunnan Province, China; c The First Clinical College, Zhejiang Chinese Medical University, Hangzhou City, Zhejiang, China; d Kunming University of Science and Technology Hospital, Kunming City, Yunnan Province, China.

**Keywords:** protocol, wound infection, Wuwei Xiaodu Drink

## Abstract

Wound infection (WI) is a disease in which pathogenic bacteria invade and multiply in a wound after trauma or surgery, causing a systemic inflammatory response. WI triggers an immune response in the body, resulting in inflammation and tissue damage, as well as slowing down the healing process. The traditional Chinese medicine prescription of Wuwei Xiaodu Drink (WWXDD) has been widely used in clinical practice with good results. However, there is no high-level evidence to support this result. The purpose of this study was to evaluate the efficacy and safety of WWXDD in the treatment of WI.

**Methods:**

We will search articles in 7 electronic databases including Chinese National Knowledge Infrastructure (CNKI), Wanfang Data (WF), Chinese Scientific Journals Database (VIP), Chinese databases SinoMed (CBM), PubMed, Embase, and Cochrane Library databases. All the publications, with no time restrictions, will be searched without any restriction on language and status, the time from the establishment of the database to October 2022. Two reviewers will independently assess the quality of the selected studies, NoteExpress and Excel software will be used to extract data, and the content will be stored in an electronic chart. Different researchers will separately screen the titles and abstracts of records acquired potential eligibility which comes from the electronic databases. Full-text screening and data extraction will be conducted afterward independently. Statistical analysis will be conducted using RevMan 5.4 software (Cochrane Collaboration).

**Results:**

What this study will do is evaluate the efficacy and safety of WWXDD in the treatment of WI in order to provide high quality, evidence-based clinical recommendations.

**Conclusion:**

This research provides a trusted clinical foundation for the treatment of WI with WWXDD.

## 1. Introduction

Trauma is a frequently encountered category in emergency medicine, which is sudden and urgent and requires rapid debridement and surgery in a short period of time. After trauma, the wound is directly exposed to air, increasing the risk of infection.^[1–3]^ Wound infection (WI) is a disease in which pathogenic bacteria invade and multiply after trauma or surgery and cause local or even systemic inflammation of the wound.^[4]^ Studies have shown that the incisional infection rate among trauma surgery patients was 13.64%, with the percentage of Gram-positive and Gram-negative bacteria and fungi being 47.37%, 39.47%, and 13.16%, respectively.^[5–7]^ Wound infections trigger the body’s immune response, causing inflammation and tissue damage, while slowing the healing process. Many minor wound infections can heal spontaneously, such as localized infections after minor skin abrasions. However, some infections can become more serious if left untreated, such as skin defects in limbs leading to amputation or even life-threatening.^[8,9]^ Rational medication is needed in the treatment of wound infections. Misuse of antibiotics not only increases the economic burden, but also easily leads to double infection. Therefore, reasonable antibiotics should be selected according to clinical drug sensitivity tests.^[10,11]^

An increasing number of scholars have observed the important value of Chinese medicine in the prevention and treatment of wound infections. Among them, Wuwei Xiaodu Drink (WWXDD) has the efficacy of clearing heat and detoxifying the toxins, reducing swelling and dispersing nodules. It is from the Qing Dynasty medical book the efficacy of clearing hea often used to prevent and treat acute surgical infections and infectious diseases. It consists of 5 herbs, namely, honeysuckle, dandelion, wild chrysanthemum, purple flowering groundnut, and sunflower seeds.^[12,13]^ Modern pharmacological studies have shown that WWXDD has a broad-spectrum antibacterial effect, especially against common pathogenic bacteria such as Staphylococcus aureus and Bacillus dysenteriae, and its angelica and astragalus can improve the immunity of the body and increase the control of inflammation, and rhizome and red peony can inhibit platelet aggregation and have anti-inflammatory and analgesic effects.^[14–17]^

Currently, majority of scholars have achieved good clinical results in treating wound infections with WWXDD, which can effectively reduce the incidence of wound infections and decrease the use of antibiotics. However, there is no evidence-based medical evidence to support the safety and efficacy of WWXDD for the treatment of WI, and the purpose of this study was to provide a reliable basis for clinical selection.

## 2. Methods

### 2.1. Study registration

This protocol report is structured according to the Preferred Reporting Items for Systematic Reviews and Meta-analysis Protocols statement.^[18]^ It is registered on the International Prospective Register of Systematic Reviews (Registration number: CRD42022370355).

### 2.2. Inclusion criteria

#### 2.2.1. Type of study

Only randomized controlled trials (RCTs) will be included irrespective of blinding, publication status, or language in this study.

#### 2.2.2. Types of participants

Patients were diagnosed with wound infection and the study belongs to a randomized controlled trial. The main outcomes included: total effective rate, antibiotics using time, white blood cell, erythrocyte sedimentation rate, C reactive protein, neutrophil, recurrence, and adverse events. The experimental group must contain WWXDD or modified WWXDD; The control group was not limited except that. Otherwise, studies will be excluded if they cannot meet the inclusion criteria.

#### 2.2.3. Types of interventions

Interventions of the experimental group are WWXDD or modified WWXDD. There are no restrictions on the way of administration, dosage, and treatment period.

#### 2.2.4. Types of control groups

The control group has other treatment methods different from WWXDD or modified WWXDD.

#### 2.2.5. Outcomes

##### 2.2.5.1. Primary outcome measures

The main outcomes is total effective rate.

##### 2.2.5.2. Secondary outcomes

The secondary outcome contains antibiotics using time, white blood cell, erythrocyte sedimentation rate, C reactive protein, neutrophil, recurrence, and adverse events.

### 2.3. Search strategy

CNKI, Wanfang, VIP, CBM, PubMed, Embase, and Cochrane Library databases were searched for this study. Take the subject terms combined with free words to search, take PubMed as an example: terms consist of (infection OR wound infection) AND (Wuwei Xiaodu Drink OR Wuwei Xiaodu Tang) AND (randomized controlled trial OR controlled clinical trial OR random trials). The searches will be conducted by two authors independently as shown in Table 1.

**Table 1:** Pubmed database search strategy.

Search number	Items
1	“Infection” [Mesh]
2	Infection [Title/Abstract]
3	Wound infection [Title/Abstract]
4	1 OR 2 OR 3
5	Wuwei Xiaodu Drink [Title/Abstract]
6	Wuwei Xiaodu Tang [Title/Abstract]
7	5 OR 6
8	Randomized controlled trial [Title/Abstract]
9	Controlled clinical trial [Title/Abstract]
10	Random trials [Title/Abstract]
11	8 OR 9 OR 10
12	4 AND 7 AND 11

### 2.4. Data collection and analysis

#### 2.4.1. Selection of studies

**Figure 1.**
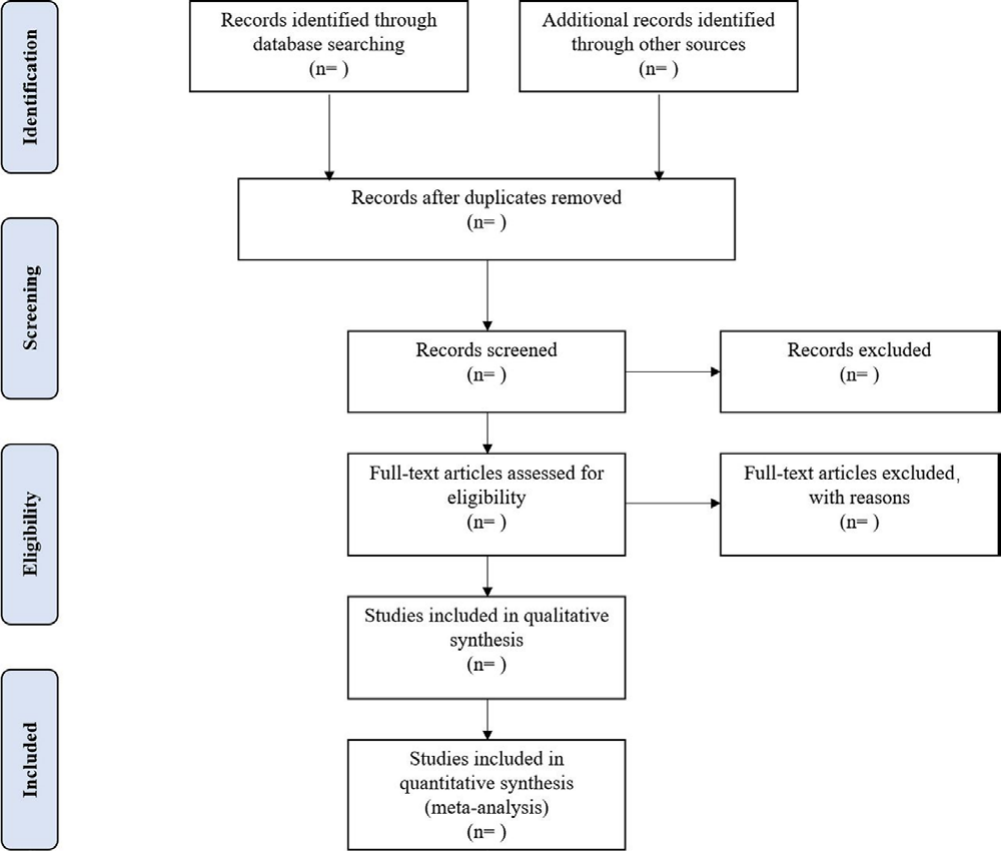
Flowchart of literature selection.

Different researchers will separately screen the titles and abstracts of records acquired potential eligibility which comes from the electronic databases. The obtained literature is managed by Notoexpress, irrelevant and duplicate articles are excluded by reading the title and abstract, full texts screening and data extraction will be conducted afterward independently, and finally selected according to the inclusion criteria, any disagreement will be resolved by discussion with the third author until consensus is reached or by consulting a third author. Preferred Reporting Items for Systematic Reviews and Meta-analysis Protocols flowchart will be used to show the selection procedure (Fig. 1).

#### 2.4.2. Data extraction and management

NoteExpress and Excel software will be used to extract data, and the content will be stored in an electronic chart. The following data will be extracted: author, year of publication, country, interventions of experimental groups and control groups, time point, outcome measures, age of patients, the total number of people included in the study, patients’ basic information, etc. Different researchers will separately extract data. Any disagreement regarding data extraction will be resolved by discussion until consensus is reached or by consulting a third author.

### 2.5. Risk of bias assessment.

Two reviewers will independently assess the quality of the selected studies according to the Cochrane Collaboration’s tool for RCTs.^[19]^ Items will be evaluated in 3 categories: Low risk of bias, unclear bias, and high risk of bias. The following characteristics will be evaluated: random sequence generation (selection bias), allocation concealment (selection bias), blinding of participants and personnel (performance bias), incomplete outcome data (attrition bias), selective reporting (reporting bias), and other biases. Results from these questions will be graphed and assessed using Review Manager 5.4. The results will be presented in the form of a graph and will be independently evaluated by 2 researchers. If there are differences of opinion, they will be discussed with the third researcher.

### 2.6. Statistical analysis

Statistical analysis will be conducted using RevMan 5.4 software (Cochrane Collaboration). For continuous data, will be used mean difference as the effect indicator with a 95% confidence interval, and dichotomous data will be calculated as risk ratio or odds ratio as the effect index with a 95% confidence interval. The *I*^2^ statistic will be used to assess levels of the heterogeneity, when *I*^2^ < 50%, the fixed-effect model can be used for analysis, otherwise, the random-effect model will be used.

### 2.7. Sensitivity analysis and subgroup analysis

We will consider the subgroup analysis intervention of the experimental group. In addition, through sensitivity analysis assess the source of heterogeneity, by excluding low-quality studies, or by excluding one of the included studies in turn, if there is no significant change in the heterogeneity, the results are robust, otherwise, the excluded study may be the heterogeneous originate.

### 2.8. Publication bias

In this study, <10 RCTs will use funnel plots to evaluate publication bias, or else, Egger’s regression test will be used.

## 3. Discussion

Numerous studies have confirmed that WWXDD is effective in reducing post-traumatic WI rates based on standard asepsis and early complete debridement, but there has been no high-level clinical evidence-based basis to convince the academic community. This study aims to address this issue and provide evidence-based evidence for clinical decision making.

## Author contributions

**Conceptualization:** Jingfan Yang, Hong Yin, Jinlei Li.

**Data curation:** Jie Luo.

**Formal analysis:** Ming Peng, Fang Liu, Jinlei Li.

**Funding acquisition:** Jinlei Li.

**Investigation:** Fang Liu, Hong Yin.

**Methodology:** Jie Luo, Xing Zhou.

**Resources:** Jie Luo.

**Software:** Ming Peng, Xing Zhou.

**Supervision:** Jingfan Yang.

**Validation:** Jingfan Yang.

**Visualization:** Ming Peng.

**Writing – original draft:** Jie Luo.

**Writing – review & editing:** Jinlei Li.
